# Supplementary figures and images for: Identification of a truncated splice variant of IL-18 receptor alpha in the human and rat, with evidence of wider evolutionary conservation
Source: PeerJ. 2014 Sep 11;2:e560. doi: 10.7717/peerj.560 (PMC4168765; doi:10.7717/peerj.560)

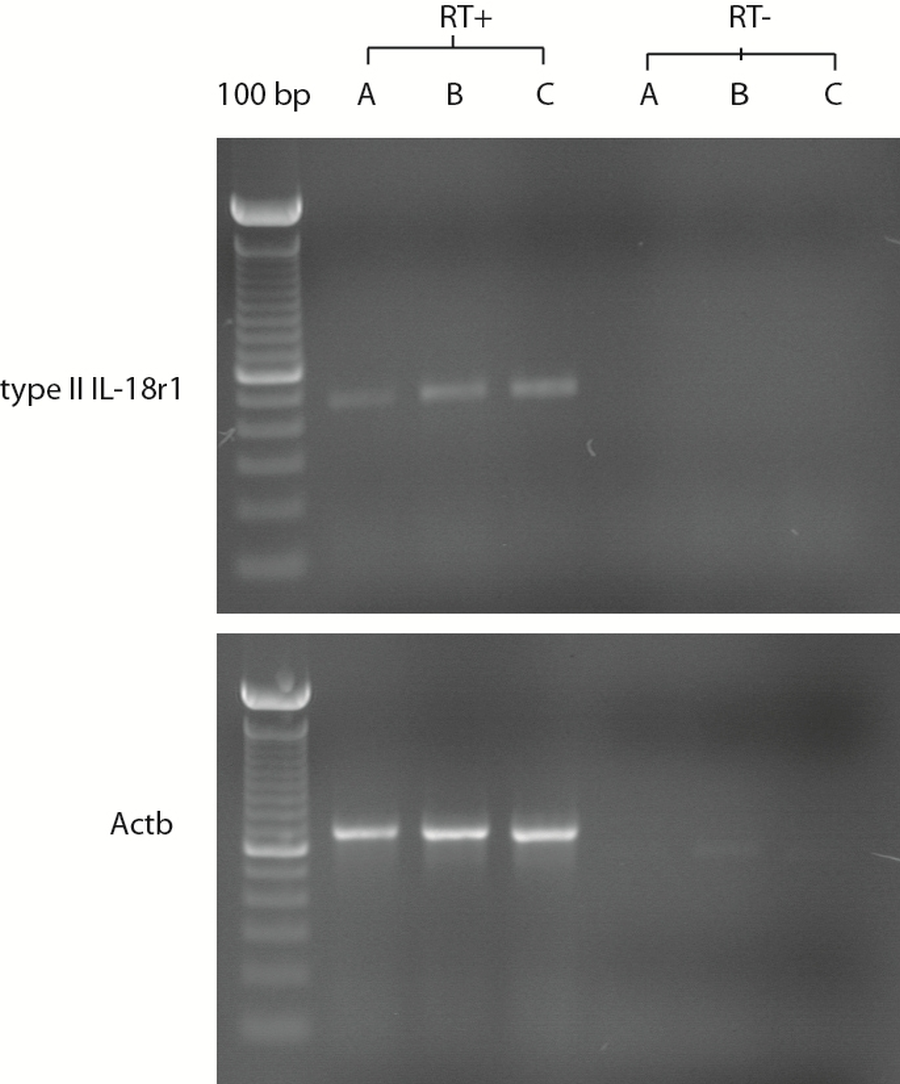

Supplement: Figure S1 — A 100 bp ladder is shown in the left lane, followed by reverse transcriptase positive (RT+) and negative (RT−) samples from 3 different rats (A)–(C). Expected product sizes for rat type II IL18r1 and Actb transcripts were 463 bp and 649 bp, respectively. [file peerj-02-560-s002.png]

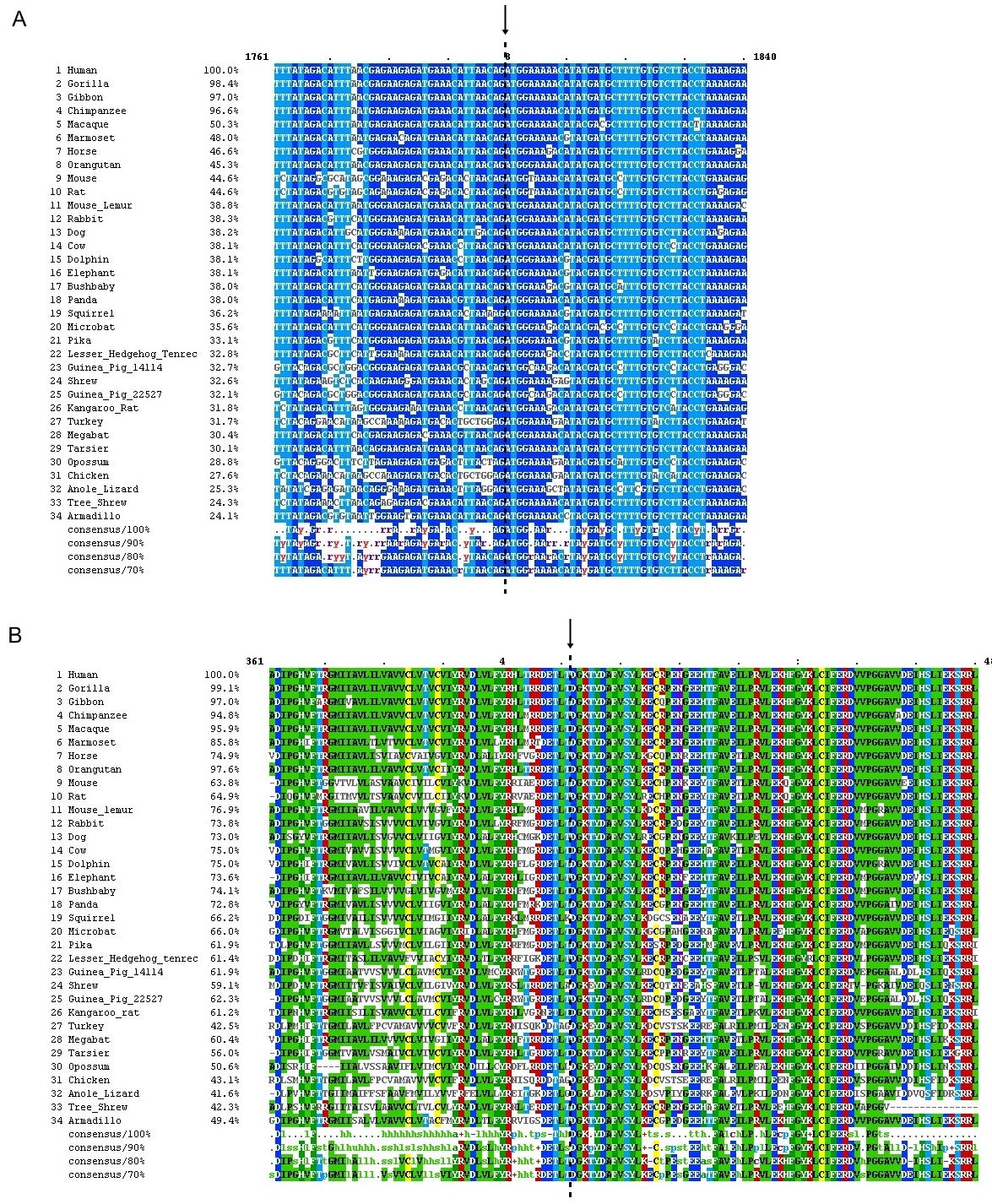

Supplement: Figure S2 — Alignments show conserved reading frames across species, the site of insertion of an unspliced intron in type II IL18R1 nucleotide sequences (arrow and dotted line in (A)), and the corresponding point in protein sequences (arrow and dotted lines in (B)) at which the more C-terminal amino acids shown would be replaced by those encoded by the unspliced intron (shown in Fig. 5). [file peerj-02-560-s003.png]
