# Supplementary material for: Identification of a truncated splice variant of IL-18 receptor alpha in the human and rat, with evidence of wider evolutionary conservation
Source: PeerJ. 2014 Sep 11;2:e560. doi: 10.7717/peerj.560 (PMC4168765; doi:10.7717/peerj.560)
Supplement: Table S1 — Note that the two identified Guinea Pig sequences differ in their 3rd Ig domain (site of IL-18 binding) and are referred to by shorthand identifiers in the text and figures: * shorthand identifier Guinea Pig 22527 (for Gene ID: ENSCPOG00000022527), † shorthand identifier Guinea Pig 14114 (Gene ID: ENSCPOG00000014114). [file peerj-02-560-s004.docx]

| **Species common name** | **Protein** | **Protein incomplete near exon/intron junction** | **Included in assessment** | **Reason for exclusion** | **Nucleotide** | **Homologous intron** | **Protein/ Transcript Name** | **Gene Name/ Description** |
| --- | --- | --- | --- | --- | --- | --- | --- | --- |
| Alpaca | ENSVPAP00000008238 | X | N | Incomplete sequence (Ns in nucleotide and/or Xs in protein sequences) |  |  | Novel | IL18R1 |
| Anole Lizard | ENSACAP00000007258 |  | Y |  | ENSACAT00000007413 | 13-14 | Novel | IL18R1 |
| Armadillo | ENSDNOP00000004245 |  | Y |  | ENSDNOT00000005475 | 7-8 | Novel | IL18R1 |
| Bushbaby | ENSOGAP00000012532 |  | Y |  | ENSOGAT00000014002 | 8-9 | Novel | IL18R1 |
| Cat | ENSFCAP00000007178 | X | N | Incomplete sequence (Ns in nucleotide and/or Xs in protein sequences) |  |  | Novel | IL18R1 |
| Chicken | ENSGALP00000027053 |  | Y |  | ENSGALT00000027104 | 10-11 | Novel | IL18R1 |
| Chimpanzee | ENSPTRP00000021076 |  | Y |  | ENSPTRT00000022838 | 7-8 | Novel | IL18R1 |
| Coelacanth | ENSLACP00000009449 | X | N | Incomplete sequence (Ns in nucleotide and/or Xs in protein sequences) |  |  | Novel | IL18R1 |
| Cow | ENSBTAP00000001371 |  | Y |  | ENSBTAT00000001371 | 8-9 | Novel | IL18R1 |
| Dog | ENSCAFP00000003126 |  | Y |  | ENSCAFT00000003368 | 8-9 | Novel | IL18R1 |
| Dolpin | ENSTTRP00000010580 |  | Y |  | ENSTTRT00000011158 | 8-9 | Novel | IL18R1 |
| Elephant | ENSLAFP00000006554 |  | Y |  | ENSLAFT00000007810 | 8-9 | Novel | IL18R1 |
| Gibbon | ENSNLEP00000017837 |  | Y |  | ENSNLET00000018727 | 8-9 | Novel | IL18R1 |
| Gorilla | ENSGGOP00000012565 |  | Y |  | ENSGGOT00000012927 | 8-9 | Novel | IL18R1 |
| Guinea Pig * | ENSCPOP00000017079 |  | Y |  | ENSCPOT00000027701 | 8-9 | Novel | novel gene |
| Guinea Pig † | ENSCPOP00000012713 |  | Y |  | ENSCPOT00000014256 | 9-10 | Novel | novel gene |
| Hedgehog | ENSEEUP00000010870 | X | N | Incomplete sequence (Ns in nucleotide and/or Xs in protein sequences) |  |  | Novel | IL18R1 |
| Horse | ENSECAP00000005479 |  | Y |  | ENSECAT00000007494 | 8-9 | Novel | IL18R1 |
| Hyrax | ENSPCAP00000010035 | X | N | Incomplete sequence (Ns in nucleotide and/or Xs in protein sequences) |  |  | Novel | IL18R1 |
| Kangaroo Rat | ENSDORP00000009207 |  | Y |  | ENSDORT00000009794 | 14-15 | IL18r1 | IL18R1 |
| Lesser Hedgehog Tenrec | ENSETEP00000014714 |  | Y |  | ENSETET00000018108 | 11-12 | Novel | IL18R1 |
| Macaque | ENSMMUP00000012112 |  | Y |  | ENSMMUT00000012921 | 8-9 | Novel | IL18R1 |
| Marmoset | ENSCJAP00000015317 |  | Y |  | ENSCJAT00000016171 | 8-9 | IL18R1 | IL18R1 |
| Megabat | ENSPVAP00000000216 |  | Y |  | ENSPVAT00000000236 | 8-9 | Novel | IL18R1 |
| Microbat | ENSMLUP00000015125 |  | Y |  | ENSMLUT00000016599 | 11-12 | Novel | IL18R1 |
| Mouse | ENSMUSP00000085298 |  | Y |  | ENSMUST00000087983 | 9-10 | IL18r1-201 | IL18R1 |
| Mouse Lemur | ENSMICP00000007990 |  | Y |  | ENSMICT00000008776 | 7-8 | Novel | IL18R1 |
| Opossum | ENSMODP00000002903 |  | Y |  | ENSMODT00000002961 | 7-8 | Novel | IL18R1 |
| Orangutan | ENSPPYP00000013510 |  | Y |  | ENSPPYT00000014059 | 8-9 | Novel | IL18R1 |
| Panda | ENSAMEP00000013892 |  | Y |  | ENSAMET00000014470 | 8-9 | Novel | IL18R1 |
| Pig | ENSSSCP00000008709 | X | N | Incomplete sequence (Ns in nucleotide and/or Xs in protein sequences) |  |  | NP_999263.1 | IL18RA |
| Pika | ENSOPRP00000005518 |  | Y |  | ENSOPRT00000006009 | 10-11 | Novel | IL18R1 |
| Rabbit | ENSOCUP00000014162 |  | Y |  | ENSOCUT00000016476 | 8-9 | B3VBV3_RABIT | B3VBV3_RABIT |
| Rat | ENSRNOP00000020194 |  | Y |  | ENSRNOT00000020194 | 9-10 | IL18r1 | IL18R1 |
| Shrew | ENSSARP00000008442 |  | Y |  | ENSSART00000009332 | 12-13 | Novel | IL18R1 |
| Sloth | ENSCHOP00000006626 | X | N | Incomplete sequence (Ns in nucleotide and/or Xs in protein sequences) |  |  | Novel | IL18R1 |
| Squirrel | ENSSTOP00000003751 |  | Y |  | ENSSTOT00000004177 | 10-11 | Novel | IL18R1 |
| Tarsier | ENSTSYP00000008004 |  | Y |  | ENSTSYT00000008717 | 10-11 | Novel | IL18R1 |
| Tasmanian Devil | ENSSHAP00000009225 |  | N | T. Devil sequences are splice variants which differ in TIR domain in similar manner as type I and type II IL18r1 |  |  | Novel | IL18R1 |
| Tasmanian Devil | ENSSHAP00000009226 |  | N | T. Devil sequences are splice variants which differ in TIR domain in similar manner as type I and type II IL18r1 |  |  | Novel | IL18R1 |
| Tree Shrew | ENSTBEP00000010624 |  | Y |  | ENSTBET00000012268 | 10-11 | Novel | IL18R1 |
| Turkey | ENSMGAP00000015699 |  | Y | Turkey transcripts similar in cytoplasmic domain, only one included in analysis | ENSMGAT00000016658 | 8-9 | Novel | IL18R1 |
| Turkey | ENSMGAP00000015697 |  | N | Turkey transcripts similar in cytoplasmic domain, only one included in analysis | ENSMGAT00000016656 |  | Novel | IL18R1 |
| Zebra Finch | ENSTGUP00000017211 |  | N | ? Functional receptor. Appears to lack extracellular domain |  |  | Novel | Uncharacterized protein |
| Zebra Finch | ENSTGUP00000010174 |  | N | ? Functional receptor. Appears to lack extracellular domain |  |  | Novel | Uncharacterized protein |
